# Geographic Distribution of Archaeal Ammonia Oxidizing Ecotypes in the Atlantic Ocean

**DOI:** 10.3389/fmicb.2016.00077

**Published:** 2016-02-09

**Authors:** Eva Sintes, Daniele De Corte, Elisabeth Haberleitner, Gerhard J. Herndl

**Affiliations:** ^1^Department of Limnology and Bio-Oceanography, Center of Ecology, University of ViennaVienna, Austria; ^2^Department of Biological Oceanography, Royal Netherlands Institute for Sea ResearchDen Burg, Netherlands

**Keywords:** Thaumarchaeota, ammonia oxidizers, ecotypes, high ammonia, low ammonia, deep ocean

## Abstract

In marine ecosystems, Thaumarchaeota are most likely the major ammonia oxidizers. While ammonia concentrations vary by about two orders of magnitude in the oceanic water column, archaeal ammonia oxidizers (AOA) vary by only one order of magnitude from surface to bathypelagic waters. Thus, the question arises whether the key enzyme responsible for ammonia oxidation, ammonia monooxygenase (amo), exhibits different affinities to ammonia along the oceanic water column and consequently, whether there are different ecotypes of AOA present in the oceanic water column. We determined the abundance and phylogeny of AOA based on their *amo*A gene. Two ecotypes of AOA exhibited a distribution pattern reflecting the reported availability of ammonia and the physico-chemical conditions throughout the Atlantic, and from epi- to bathypelagic waters. The distinction between these two ecotypes was not only detectable at the nucleotide level. Consistent changes were also detected at the amino acid level. These changes include substitutions of polar to hydrophobic amino acid, and glycine substitutions that could have an effect on the configuration of the amo protein and thus, on its activity. Although we cannot identify the specific effect, the ratio of non-synonymous to synonymous substitutions (dN/dS) between the two ecotypes indicates a strong positive selection between them. Consequently, our results point to a certain degree of environmental selection on these two ecotypes that have led to their niche specialization.

## Introduction

Nitrogen is fundamental for all living organisms and is present in the environment in a variety of organic and inorganic forms. Microorganisms play a key role in the nitrogen cycle as they can conduct all the different processes involved in the transformation from one form of nitrogen to another. Nitrification, the oxidation of ammonia to nitrite that can be subsequently oxidized to nitrate, is an important step in this cycle. Microbial ammonia oxidation was thought to be restricted to Bacteria (Ward et al., 2007), however, the capability of Archaea to conduct this process was first suggested a decade ago, after the gene encoding for ammonia monooxygenase was discovered in Archaea (Venter et al., 2004). Marine Archaea are dominated by mesophilic Marine Group I Crenarchaeota, recently coined Thaumarchaeota (Brochier-Armanet et al., 2008; Spang et al., 2010). The first isolate of Thaumarchaeota was shown to oxidize aerobically ammonia to nitrite (Könneke et al., 2005). Since then, archaeal ammonia oxidizers (AOA) were found to dominate in many terrestrial and marine ecosystems over ammonia oxidizing bacteria (AOB) including the deep ocean (Treusch et al., 2005; Wuchter et al., 2006; Agogué et al., 2008). The out-competition of Bacteria by Archaea in specific environments was proposed to be driven by chronic energy stress (Valentine, 2007), and in particular in the case of archaeal nitrifiers, they were proposed to out-compete Bacteria in conditions of low energy availability such as in the oligotrophic ocean. This hypothesis was supported by the finding of a lower half saturation constant (Km) and substrate threshold for AOA (Martens-Habbena et al., 2009; Horak et al., 2013) as compared to AOB (Stark and Firestone, 1996), with important implications for the nitrogen cycle in the ocean (Martens-Habbena et al., 2009), where ammonia concentration is generally lower than the requirements of AOB.

However, marine AOA are not a homogenous group. Two main clusters (Francis et al., 2005) have been suggested to represent vertically segregated groups in marine environments (Hallam et al., 2006). The relationship between ammonia oxidation rates and the abundance of genes from these two (surface vs. deep) clusters were explored in the Gulf of California (Beman et al., 2008) and Monterey Bay (Smith et al., 2014) indicating no or only a small contribution to the measured nitrification rates in surface waters of the “deep cluster.” Recently, we showed that two phylogenetically distinct clusters of marine AOA inhabit different depth layers and regions characterized by contrasting ammonia availability (Sintes et al., 2013). Consequently, AOA were divided into the high ammonia concentration AOA (HAC-AOA) dominating in regions of the ocean with relatively high ammonia concentrations, such as the Arctic and epipelagic waters, roughly corresponding to the surface AOA cluster. In contrast, the low ammonia concentration AOA (LAC-AOA) dominate in deep-ocean environments with ammonia concentrations below the detection limit of conventional methods (Sintes et al., 2013). Studies on tetraether lipid pathways from Thaumarchaeota in the Arabian Sea also indicate that ammonia availability plays an important role in the distribution pattern of AOA ecotypes (Villanueva et al., 2015). The existence of AOA ecotypes adapted to different ammonia supply rates might have major biogeochemical and ecological implications, similarly to the realization of the key role of AOA as drivers of the nitrification processes in the ocean (Mincer et al., 2007; Church et al., 2010; Santoro et al., 2013). Up to now, only members of the HAC cluster have been isolated or enriched, mainly belonging to the *Nitrosopumilus* genus (Könneke et al., 2005; Santoro and Casciotti, 2011; Park et al., 2014; Bayer et al., 2016), and *Nitrosopelagicus brevis* (Santoro et al., 2015), with reported Km values of 65–133 nM for isolates and open ocean waters. Thus, the existence of the LAC ecotype adapted to low ammonia concentrations would suggest that AOA might be able to use ammonia as energy source at lower concentrations than the present isolates, with important implications for the nitrogen cycle especially in the deep ocean and the oligotrophic tropical and subtropical oceans.

In community ecology, competition between organisms plays an important role in selection. The competitive exclusion principle (Gause, 1934; Hardin, 1960) indicates that the selection of organisms can be driven by resource limitation. According to this principle, in classic ecology, if two organisms compete for the same resource, the one less fitted will be out-competed by the other one. However, the species could continue coexisting if adaptation to slightly different conditions, and hence, niche segregation occurs. Thus, we hypothesized that the diversification into the HAC-AOA and LAC-AOA cluster results from the competition for resources and selective pressure.

The goal of this study was to provide a comprehensive view on the niche specialization of two distinct functional groups of AOA and their distribution along a latitudinal gradient (from 65°N to 55°S) and throughout the water column of the Atlantic Ocean. In order to do that, we used a similar approach as in Sintes et al. (2013) where we described these two ecotypes in two contrasting environments (coastal Arctic and tropical Atlantic). The distribution of the two groups assessed via q-PCR of their *amo*A gene will be evaluated in different oceanographic regions characterized by different environmental and biological conditions (Longhurst, 2007). The results should complement and expand our knowledge on the distribution of AOA ecotypes in the ocean. Based on our previous findings, we hypothesized that the differentiation of AOA ecotypes inhabiting the different depth layers including the bathypelagic realm results from selective pressure on the substrate affinity of the ammonia monooxygenase which, in turn, should be reflected in the *amo*A gene. Using the same datasets of 454-pyrosequenced and Sanger sequenced *amo*A from Sintes et al. (2015), where we explored macroecological patterns of AOA at the community level, translated gene oligotyping will be used to support the ecotype differentiation at the protein level.

## Materials and methods

**Figure 1:**
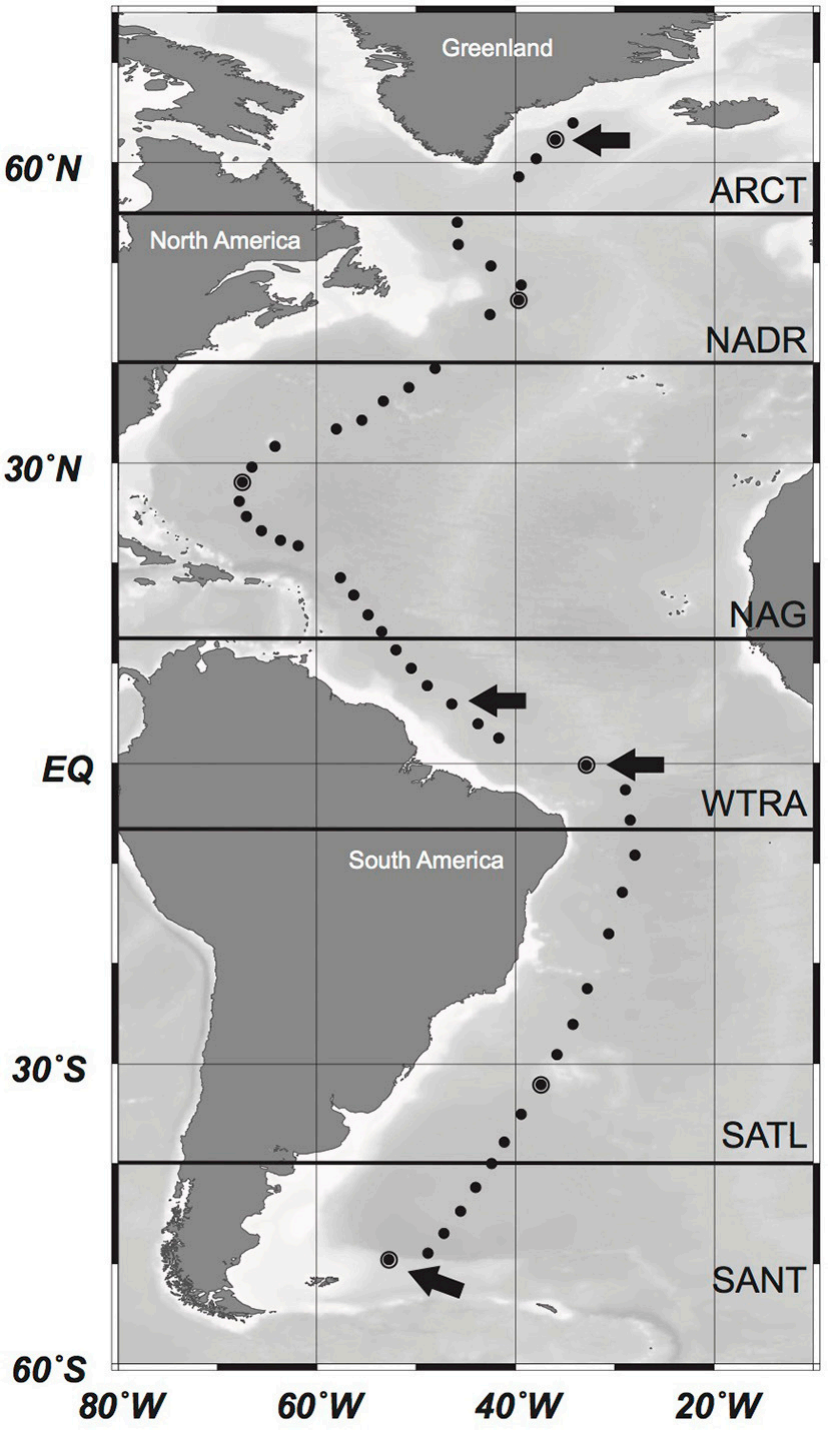
**Location of the sampling stations along the cruise track in the Atlantic Ocean**. Oceanographic regions based on Longhurst (2007) are indicated by bold horizontal lines. ARCT, North Atlantic Arctic Province; NADR, North Atlantic Drift Province; NAG, North Atlantic Gyral Province; WTRA, Western Tropical Atlantic; SATL, South Atlantic Gyral Province; SANT, Subantarctic Province. Pyrosequencing was conducted in encircled stations. Stations where additionally cloning and sequencing of the *amo*A gene was conducted are marked with an arrow. Modified from Sintes et al. (2015).

Sampling was conducted during the GEOTRACES-1 to -3 cruises on board R/V *Pelagia* and R/V *James Cook*, between April and June 2010 and between February and April 2011, respectively. Water samples were taken at 51 stations (Figure 1) with 25L-Niskin bottles mounted in a frame holding also sensors for conductivity-temperature-depth (CTD), salinity, oxygen, fluorescence, and optical backscattering. Samples for analyses of inorganic nutrients, trace elements, and microbial abundance were collected at 24 depth layers from surface to abyssopelagic waters as detailed below. Samples for the analyses of AOA were collected at 6–8 depths from the euphotic layer (50 m depth) to the lower bathypelagic and abyssopelagic depths (>2000 m). Six different oceanographic regions were differentiated along this transect based on the description of biogeographic ocean provinces (Longhurst, 2007): the North Atlantic Arctic province (ARCT; 70°N–55°N), the North Atlantic Drift province (NADR; 55°N–40°N), the North Atlantic Gyral province (NAG) comprising the North Atlantic Tropical and the Subtropical Gyral province (40°N–12°N), the Western Tropical Atlantic (WTRA; 12°N–6°S) province, the South Atlantic Gyral (SATL; 6°S–40°S), and the Subantarctic province (SANT) comprising the Subtropical Convergence Zone (SSCT; 40°S–45°S) and the Subantarctic Water Ring province (SANT; 45°S–55°S) (Figure 1).

### Inorganic nutrient concentrations

The concentrations of dissolved inorganic nutrients (NO${}_{3}^{-}$, NO${}_{2}^{-}$, PO${}_{4}^{3-}$) were determined onboard immediately after collection on 0.2 μm filtered water samples in a TRAACS 800 autoanalyzer system (Reinthaler et al., 2008).

### DNA extraction

Depending on the sampling depth, 2 (epipelagic) to 10 L (meso- to abyssopelagic) of seawater were filtered through 0.22 μm GTTP polycarbonate filters (Millipore). Subsequently, the filters were stored at -80°C until processing in the home laboratory. The extraction was performed using Ultraclean soil DNA isolation kit (Mobio). The DNA concentration in the extracts ranged between 2 and 25 ng μL^−1^ (average 8 ng μL^−1^).

### Quantitative PCR

Quantitative PCR (q-PCR) was used to evaluate the abundance of the 16S rRNA gene of Thaumarchaeota, the “low-ammonia concentration” archaeal *amo*A (LAC-archaeal *amo*A) and the “high-ammonia concentration” archaeal *amo*A (HAC-archaeal *amo*A) genes as previously described (Sintes et al., 2015) using specific primers (Table 1). The primers GI-751F and GI-956R (Mincer et al., 2007) were chosen as compared to the previously used primers MCGI-391F and MCGI-554R (Coolen et al., 2007), due to the reported underestimation of Thaumarchaeal 16S rRNA genes in some environments with this set (Alonso-Sáez et al., 2012).

**Table 1:** **Primer sets and annealing temperatures used for Thaumarchaeota 16S rRNA gene and archaeal *amo*A PCR amplification**.

**Target**	**Analysis**	**Primer name**	**Primer sequence 5′ → 3′**	**Annealing (°C)**	**Fragment (bp)**	**References**
Marine crenarchaeota	Q-PCR	GI-751F	GTCTACCAGAACAYGTTC	58	205	Mincer et al., 2007
		GI-956R	HGGCGTTGACTCCAATTG			Mincer et al., 2007
HAC-*amo*A	Q-PCR	Arch-*amo*A-for	CTGAYTGGGCYTGGACATC	59	256	Wuchter et al., 2006
		Arch-*amo*A-rev	TTCTTCTTTGTTGCCCAGTA			Wuchter et al., 2006
LAC-*amo*A	Q-PCR	Arch-*amo*A-for	CTGAYTGGGCYTGGACATC	59	256	Wuchter et al., 2006
		Arch-*amo*A-rev-New	TTCTTCTTCGTCGCCCAATA			Sintes et al., 2013
Total archaeal *amo*A	Cloning/454	cren amo_F	ATGGTCTGGCTAAGACGMTGTA	55	632	Hallam et al., 2006
		amoAR	GCGGCCATCCATCTGTATGT			Francis et al., 2005

Q-PCR analysis was performed at all 51 stations and at 6–8 depths per station. All q-PCR analyses were performed on a LightCycler 480 thermocycler (Roche) equipped with LightCycler 480 gene scanning software (version 1.5, Roche). The abundance of the thaumarchaeal 16S rRNA gene, LAC-archaeal *amo*A, and HAC-archaeal *amo*A were determined in triplicate on the non-diluted sample. The “total” archaeal *amo*A gene abundance was calculated as the sum of LAC- and HAC-archaeal *amo*A gene abundance. The reaction mixture (10 μL) contained 1 × LightCycler 480 DNA SYBRGreen I Master (Roche), 0.2 μM of primers, 1 μL of DNA extract, and was made-up to 10 μL with PCR-grade water (Roche). All reactions were performed in 96-well q-PCR plates (Roche) with optical tape. Accumulation of newly amplified double stranded gene products was followed online as the increase of fluorescence due to the binding of the fluorescent dye SYBRGreen®. Specificity of the q-PCR reaction was tested on agarose gel electrophoresis and with a melting curve analysis (65–95°C) to identify unspecific PCR products. Each gene fragment was detected using a standard and primer combinations and annealing temperature for the specific quantification of the different genes as detailed in Table 1. Thermocycling was performed as follows for the LAC- and HAC-AOA: initial denaturation at 95°C for 10 min; amplification: 50 cycles, at 95°C for 5 s, primer annealing temperature for 5 s, and extension at 72°C for 15 s, 80°C for 3 s, with a plate read between each cycle; melting curve 65–95°C with a read every 0.2°C held for 1 s between each read. Thermocycling for 16S rRNA gene was: initial denaturation at 95°C for 10 min; amplification: 50 cycles, at 95°C for 5 s, primer annealing temperature for 15 s, and extension at 72°C for 25 s, 78°C for 3 s, with a plate read between each cycle, and melting curve similar to the AOA. Efficiency of the reaction was 84.7 ± 2.4% for 16S rRNA, 91.2 ± 7.4% for LAC-AOA, and 84.5 ± 3.9% for HAC-AOA.

### Cloning, sequencing, and phylogenetic analysis of archaeal *amoA*

The full-length archaeal *amo*A from epipelagic, mesopelagic, upper and lower-bathypelagic depth layers from four different stations (Figure 1) was amplified using the primers cren amo_F (Hallam et al., 2006) and amoAR (Francis et al., 2005) (Table 1). Thermocycling was performed as follows: initial denaturation at 94°C for 4 min; amplification: 35 cycles, at 94°C for 1 min, 55°C for 1 min, and extension at 72°C for 1 min, followed by a final extension step at 72°C for 7 min and holding at 4°C. The PCR product was purified using PCRExtract MiniKit (5-PRIME) and cloned with the TOPO-TA cloning kit® (Invitrogen) according to the manufacturer's instructions. Clones were checked for the right insert by running the PCR product on a 2% agarose gel. Sequencing was performed by MACROGEN Europe using the M13 primers. The sequence data from a total of 971 clones were compiled using MEGA-5 software, and aligned together with environmental archaeal *amo*A sequences, and full-length sequences of *amo*A genes from *Nitrosopumilus maritimus, Candidatus Nitrosopumilus adriaticus, Candidatus Nitrosopelagicus brevis, Candidatus Nitrosoarchaeum limnia, Candidatus Cenarchaeum symbiosum, Candidatus Nitrososphaera gargensis*, and *Candidatus Nitrosocaldus yellowstonii* obtained from the NCBI database. Sequences from water column clusters A and B (Francis et al., 2005) were also included for reference. Operational taxonomic units (OTUs) were defined as a group of sequences differing by < 2%, resulting in 251 *amo*A sequences. Phylogenetic analyses were conducted in MEGA-5 (Tamura et al., 2007). The evolutionary history was inferred using the Neighbor-Joining method (Saitou and Nei, 1987). The bootstrap consensus tree inferred from 1000 replicates was taken to represent the evolutionary history of the taxa analyzed (Felsenstein, 1985). Branches corresponding to partitions reproduced in <50% bootstrap replicates were collapsed. The tree was drawn to scale, with branch lengths in the same units as those of the evolutionary distances used to infer the phylogenetic tree. The evolutionary distances were computed using the Maximum Composite Likelihood method (Tamura et al., 2004) and are in the units of the number of base substitutions per site. All positions containing gaps and missing data were eliminated from the dataset (complete deletion option). Phylogenetic trees were drawn using iTOL (Letunic and Bork, 2007).

454-pyrosequencing of archaeal *amo*A was performed at IMGM Laboratories GmbH (Germany) on a Roche 454 GS Junior platform based on titanium chemistry as previously described (Sintes et al., 2015). All samples were barcoded using multiplex identifiers and sequenced together in one run. Processing of the sequences was performed as described elsewhere (Sintes et al., 2015). Briefly, raw 454-sequences were initially trimmed using Lucy 1.20 (Chou and Holmes, 2001) keeping sequences of ≥250 nt which had an average Phred score of ≥27. Subsequently, the remaining sequences were screened for the barcode and primer sequences keeping only the sequences that had exact matches. The sequences selected by the above procedure were processed following a similar pipeline as described elsewhere (Pester et al., 2012). AOA OTUs were assigned as the gene sequences sharing 98% identity (Agogué et al., 2008).

Oligotype analysis (Eren et al., 2013) was conducted on the Sanger and the pyro-sequenced *amo*A gene using the oligotyping pipeline (available at http://merenlab.org/2012/05/11/oligotyping-pipeline-explained). The *amo*A gene sequences were aligned to the most recent AOA database (Pester et al., 2012). Subsequently, the pyrosequences were trimmed to a length of 240 bp to fulfill the requirements of the oligotype pipeline, while the whole length *amo*A gene sequence from Sanger sequencing (~630 bp) was used for the analysis. The entropy analysis identified 23 and 12 high entropy positions for Sanger- and 454-pyrosequencing-derived *amo*A libraries (NGS, Next Generation Sequencing). Additionally, the oligotype pipeline was applied to the translated and aligned amino acid sequences obtained from the Sanger or 454-pyrosequencing approaches. Twenty-one and 11 high entropy positions were identified at the protein level for Sanger and NGS libraries, respectively. To minimize the noise, oligotypes that had not a minimum percent abundance larger than 1% in at least one sample or had a substantial abundance below one for the pyrosequencing data were removed.

The ratio of non-synonymous to synonymous substitutions (dN/dS) was calculated using KaKs calculator (Zhang et al., 2006) for paired *amo*A sequences and Hyphy (Pond et al., 2005) for the Sanger dataset.

Sequence information used in this study was deposited in Genbank, accession numbers KF727022-KF727275. Raw 454-pyrosequences were submitted to the Sequence Read Archive (SRA) at NCBI under the Accession number SRP049002.

### Statistical analysis

Relationships between AOA variables (abundance of the HAC and LAC ecotypes, and ratio LAC/HAC), and environmental variables were analyzed using the Distance Based Linear Model (DISTLM) (Anderson et al., 2004) in Primer v6 (PRIMER-E) with an implementation of distance based redundancy analysis (dbRDA). Forward selection and adjusted *r*^2^ criterion were used to select the predictor variables and the model that best fit the data. Environmental variables were normalized, and Bray-Curtis similarities were calculated for the AOA variables. Environmental variables included latitude (absolute), depth, temperature, salinity, fluorescence, dissolved oxygen concentration, total alkalinity, dissolved inorganic carbon (DIC), macronutrients (phosphate, silicate, and nitrite), and trace elements (Al, Cd, Fe, Mn, Ni, Pb, Zn, Y, La). Only the environmental variables that were measured throughout the Atlantic were evaluated. Nitrate was excluded from the analysis due to covariation with phosphate (Weber and Deutsch, 2010). Samples with missing values were eliminated, resulting in a final set of 349 samples. The dataset of environmental data and the methods used for the measurements are available at the Geotraces website (http://www.geotraces.org) (Mawji et al., 2015).

## Results

### Latitudinal trends of marine thaumarchaeota and the abundance of archaeal ammonia oxidizers in the atlantic ocean

**Figure 2:**
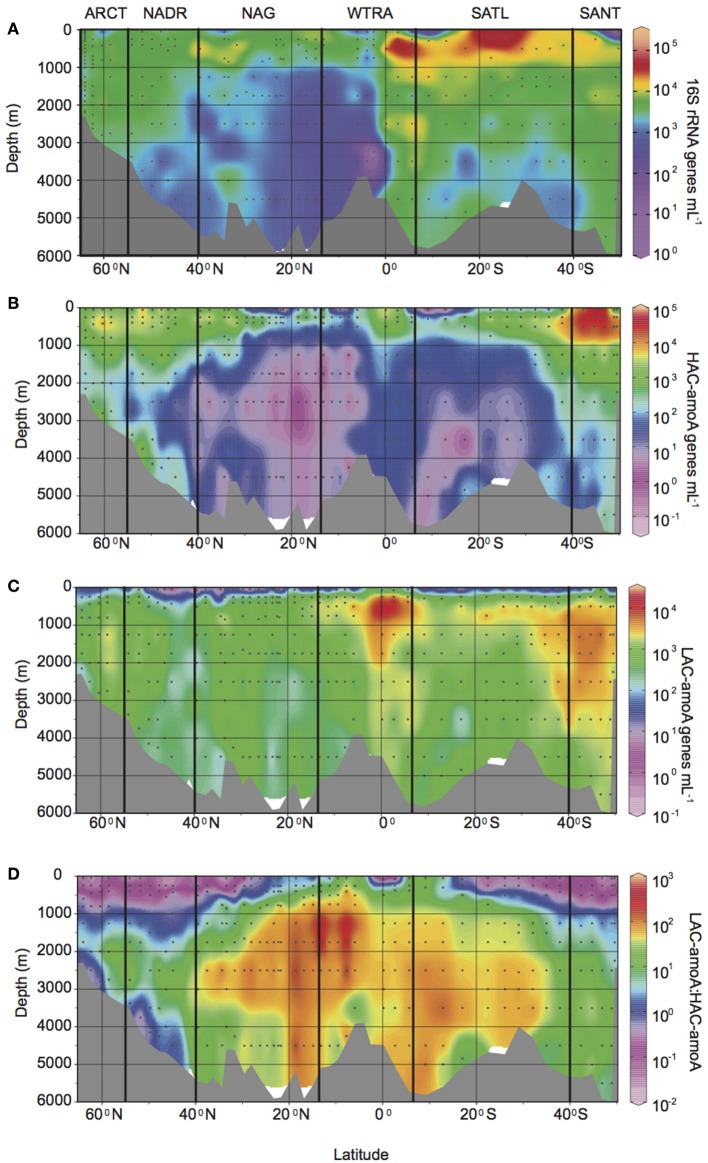
**Gene abundance (genes mL^−1^) throughout the Atlantic Ocean determined by q-PCR: of (A) thaumarchaeal 16S rRNA gene, (B) HAC-*amo*A and (C) LAC-*amo*A gene, (D) ratio between archaeal LAC-*amo*A and HAC-*amo*A gene abundance**. Bold vertical lines denote borders between oceanic provinces based on the description given in Longhurst (2007) as detailed in Figure 1.

The abundance of Thaumarchaeota measured as 16S rRNA gene abundance by q-PCR, was highest in the oxygen minimum layers throughout the Atlantic Ocean (Figure 2A, Supplementary Table 1), with an average of 1.3 ± 1.8 × 10^4^ genes mL^−1^ vs. an average gene abundance of 1.8 − 5.3 × 10^3^ genes mL^−1^ in other depths layers. Also, relatively higher abundances of the 16S rRNA gene were found in upper and lower bathypelagic environments at high latitudes (ARCT, NADR, and SANT provinces, with average gene abundance ranging from 2.0 − 7.4 × 10^3^ genes mL^−1^) and at the equator (on average 0.5 − 1.1 × 10^4^ genes mL^−1^) than in the gyre regions (NAG and SATL provinces with average gene abundance of 1.3 − 6.7 × 10^3^ genes mL^−1^). A similar distribution pattern was observed for total *amo*A gene abundance, resulting in a highly significant correlation between total *amo*A and 16S rRNA gene abundance of Thaumarchaeota (log AOA = 0.82 + 0.84 log 16S rRNA, *r*^2^ = 0.7, *p* < 0.001, *n* = 351). High ammonia concentration (HAC)-*amo*A gene abundance (Figure 2B) showed a more pronounced decrease with depth, especially at lower latitudes (NAG, SATL, and WTRA provinces) than thaumarchaeal 16S rRNA and low ammonia concentration (LAC)-*amo*A gene abundance (Figure 2C). HAC gene abundance decreased from average values ranging between 0.7 − 6.1 × 10^3^ genes mL^−1^ in epi- and mesopelagic waters down to 10–52 genes mL^−1^ in upper and lower bathypelagic waters at lower latitudes (Figure 2, Supplementary Table 1), with the exception of the epipelagic from SATL where on average 25 ± 22 genes mL^−1^ were observed.

Thus, putatively different functional groups of AOA dominated the thaumarchaeal communities in different regions and depth layers (Figure 2D, Supplementary Table 1). The HAC-*amo*A gene dominated the AOA community in epi- and mesopelagic waters at high latitude regions (Figure 2D, average ratio LAC/HAC ranging between 0.02 and 0.36). In contrast, LAC-*amo*A dominated the bathy- to abyssopelagic waters throughout the Atlantic (average ratio LAC/HAC 47 ± 71) and the mesopelagic realm at low latitude regions (Figure 2D) with LAC/HAC ratios ranging from 4 to 38 in SATL and WTRA, respectively.

### Diversity of archaeal ammonia oxidizers: latitudinal and depth patterns

**Figure 3:**
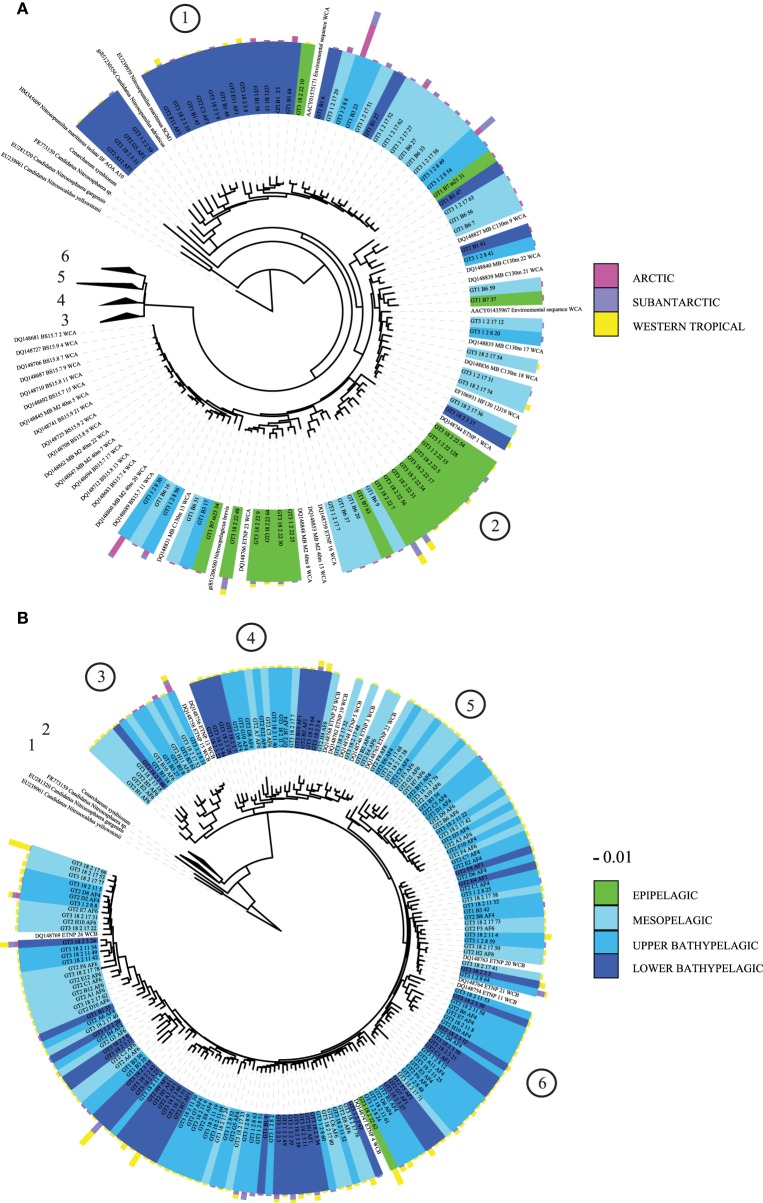
**Phylogenetic tree of archaeal *amo*A sequences recovered from the Atlantic: (A) expanded view for subclusters 1 and 2, (B) expanded view for subclusters 3–6**. Reference sequences of *Nitrosopumilus maritimus, Nitrosopumilus adriaticus, N. brevis, Nitrososphaera gargensis, Cenarchaeum symbiosum*, and *Nitrosocaldus yelowstonii* are indicated. Several sequences from water cluster A (surface) and B (deep) clusters (Francis et al., 2005; Mincer et al., 2007) are also indicated. Green: epipelagic, light to dark blue tones: mesopelagic (250 m) to lower bathypelagic (~6000 m depth). One representative of sequence group >98% identical is shown; the bar shows the number of clones represented by a sequence, and the colors indicate the oceanographic region (ARCT, SANT, and WTRA). Encircled numbers indicate the six main subclusters identified in the text.

From the five clusters of AOA previously identified (Pester et al., 2012), cloning and sequencing of the archaeal *amo*A gene revealed six main subclusters branching to the *N. maritimus* cluster (Figure 3). Four of these subclusters were dominated by sequences from deep-waters (>200 m depth) with a high OTU richness in the WTRA province (subclusters 3–6, Figure 3B). Most epipelagic archaeal *amo*A sequences affiliated to subcluster 2 (Figure 3A), while subcluster 1 included bathypelagic sequences from several provinces clustering with *N. maritimus amo*A (Figure 3A). Subclusters 1 and 2 include the sequences from Water Cluster A (WCA) or surface cluster (Francis et al., 2005; Mincer et al., 2007). The HAC-primers, allowing one mismatch, targeted all the sequences of subclusters 1 and 2 (Figure 3A), but just 5 out of 509 clones of subclusters 3–6. The LAC-primers targeted only sequences of subclusters 3–6 (Figure 3B), which include sequences from Water Cluster B or deep cluster (Francis et al., 2005; Hallam et al., 2006; Mincer et al., 2007). Unifrac analysis of significance on the AOA community composition assessed by Sanger sequencing indicated distinct differences between provinces and depth layers (*p* < 0.001).

### Ecotypes of archaeal ammonia oxidizers

**Figure 4:**
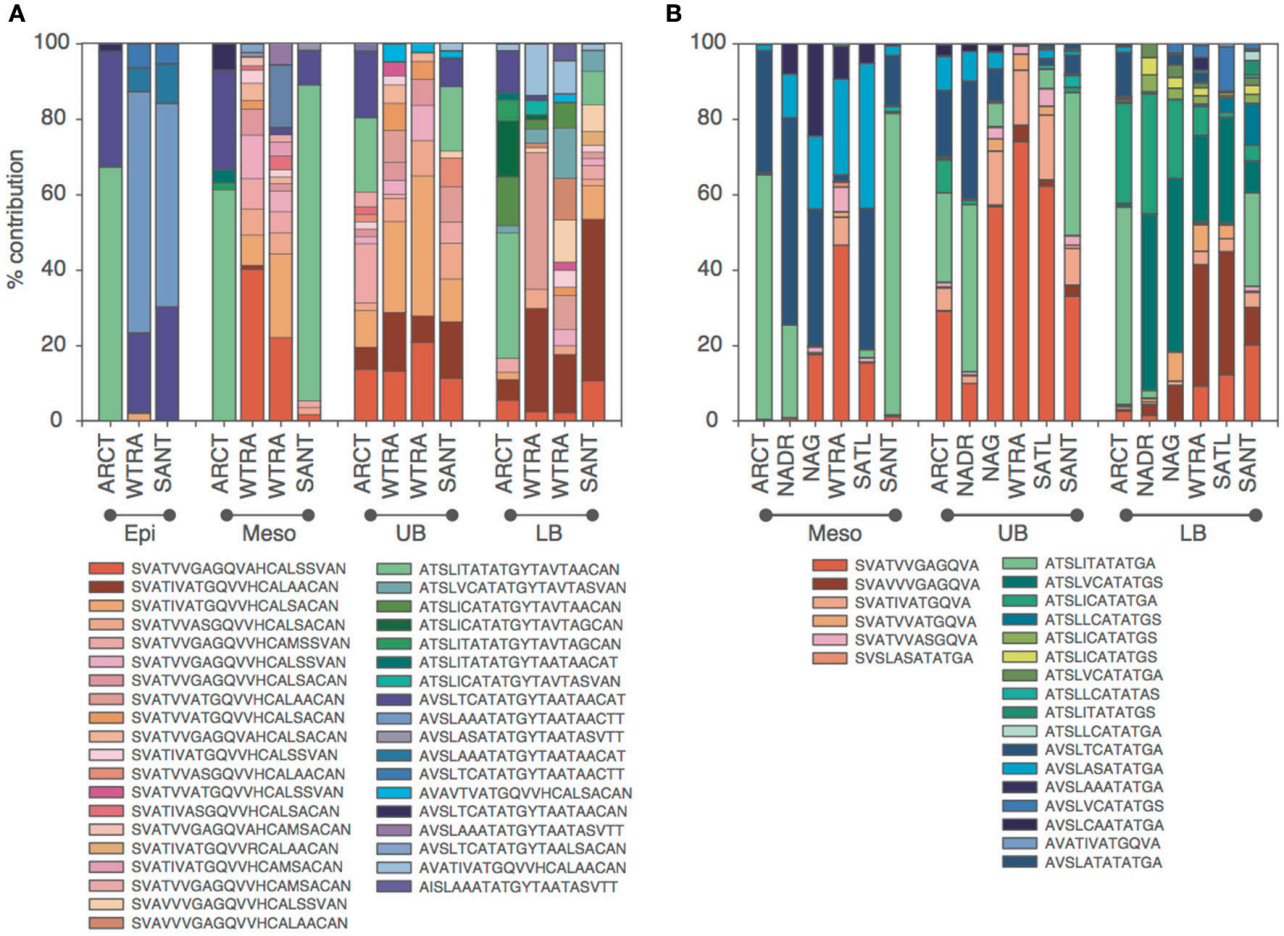
**Distribution of the oligotypes obtained from translated *amo*A Sanger (A) and NGS (B) libraries in different regions for distinct depth layers (Epi, epipelagic; Meso, mesopelagic; UB, upper bathypelagic; LB, lower bathypelagic)**. Red and green/blue colors indicate *amo*A with Serine and Alanine in position 19, respectively. Green and blue colors indicate Threonine and Valine (Isoleucine) at position 34, respectively.

**Figure 5:**
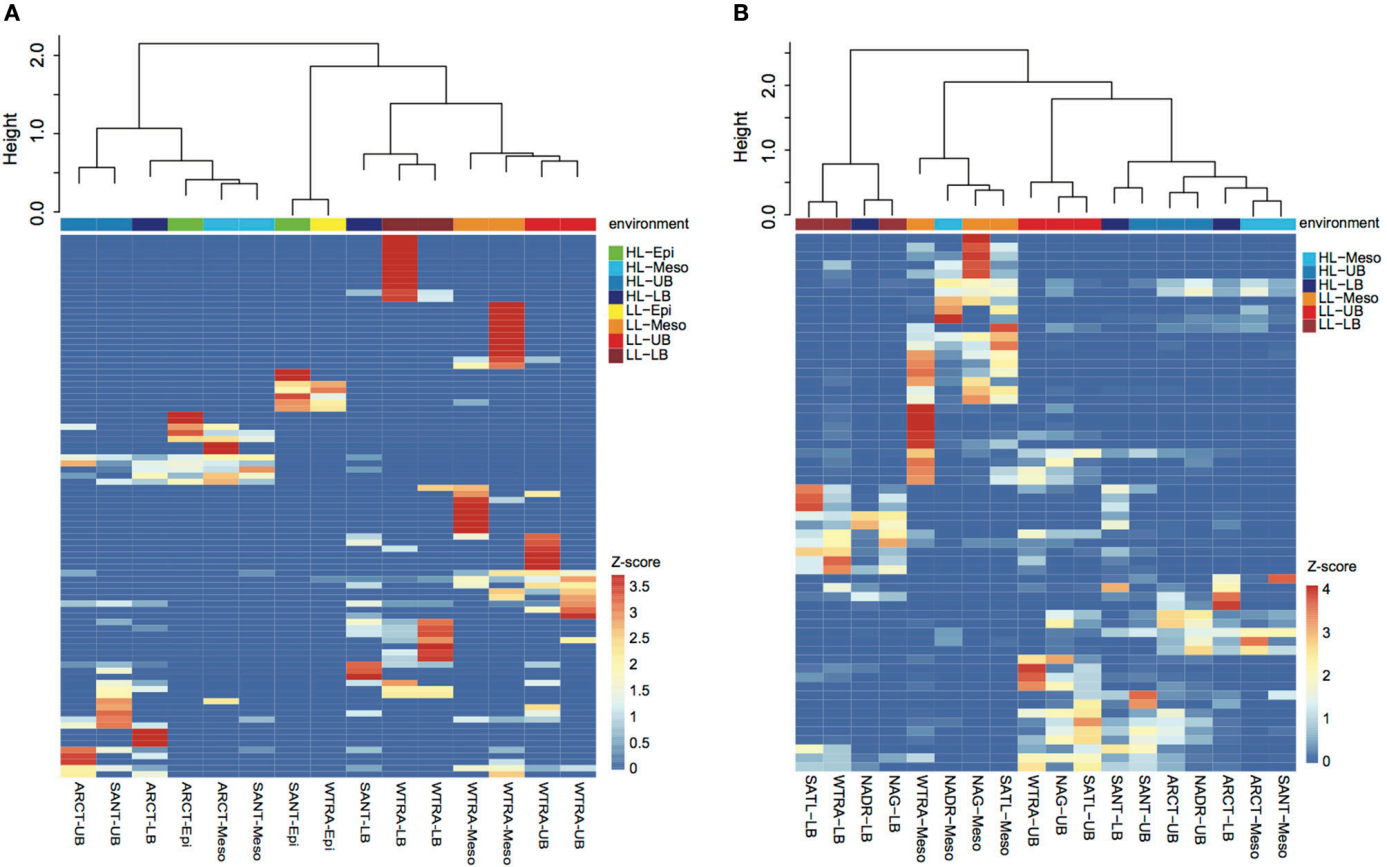
**Heatmap visualizing the z-score distribution of the different oligotypes obtained by Sanger sequencing (A) and pyrosequencing (B) among the samples collected in the Atlantic Ocean**. The dendogram clusters samples according to the Bray-Curtis similarity index. Samples are colored according to the environment, HL, high latitude (>40°); LL, low latitude (< 40°); and depth layer.

**Figure 6:**
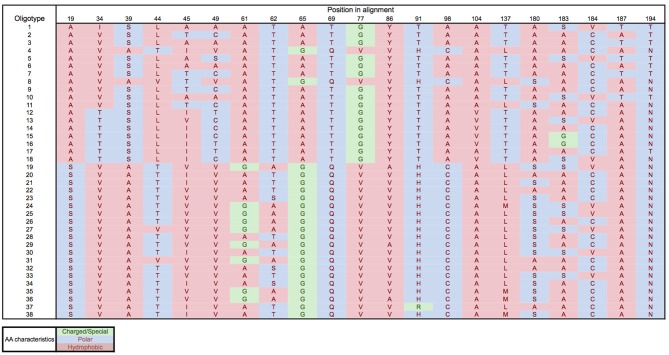
**Amino acid residues for the different oligotypes obtained from Sanger sequenced *amo*A**. Amino acids: A, Alanine; S, Serine; V, Valine; T, Threonine; L, Leucine; I, Isoleucine; C, Cysteine; G, Glycine; Q, Glutamine; Y, Tyrosine; H, Histidine; R, Arginine; M, Methionine; N, Asparagine.

Oligotyping of the *amo*A gene at the nucleotide level identified 89 and 60 oligotypes according to Sanger sequenced full-length AOA and 454-pyrosequencing, respectively. Oligotyping at the amino acid level differentiated 38 and 22 oligotypes from Sanger and NGS sequencing libraries (Figure 4) with varying amino acid residues at 21 and 11 positions, respectively. Few oligotypes contributed a large fraction of the archaeal community oligotypes from specific regions or from specific depth layers, both at the nucleotide and amino acid level (Figures 4, 5). Two main groups could be distinguished according to changes between hydrophobic and polar or charged residues (Figure 6). Oligotypes with serine (Ser) at position 19 dominated in deep waters and low latitudes and were targeted by the LAC-amoA primers (Figure 4). Oligotypes with alanine (Ala) at this same position dominated in epipelagic and high latitudinal regions (Figure 4) and were targeted by the HAC-*amo*A primers. These two groups of oligotypes had consistent changes between non-synonymous amino acids at another seven positions (with amino acids changes present in ≥75% of the individual oligotypes from the group): positions 39, 44, 65, 77, 98, 137, 180 (Figure 6). The distribution of the distinct oligotypes resulted in a clustering of the AOA communities according to latitude (high >40°N or S, vs. low 0–40°), and a subclustering according to depth (Figure 5).

### Environmental factors explaining the distribution of ecotypes

Most of the available environmental variables significantly explained the variation of the AOA ecotypes (Table 2). The percent variation explained by the significant variables ranged between 55 and 65% for the HAC-AOA abundance and the ratio LAC/HAC, respectively. Explanatory variables common to the three AOA parameters were, depth, temperature, fluorescence, DIC, Cd, Fe, Mn, Pb, Y, and La. Latitude, salinity, oxygen, Ni, and phosphate concentration contributed to explain the variation of HAC abundance as well as the ratio LAC/HAC. Nitrite concentration contributed to explain the variation in LAC-AOA and in the ratio LAC/HAC.

**Table 2:** **Distance-based linear model (DISTLM) results of the best set of environmental variables that account for variations in HAC-AOA, LAC-AOA, and ratio LAC/HAC for the whole data (*n* = 349)**.

	**HAC mL**^**−1**^				**LAC mL**^**−1**^				**LAC/HAC**			
**Variables**	**Prop**.	**Pseudo-F**	***p***	**Coeff**	**Prop**.	**Pseudo-F**	***p***	**Coeff**	**Prop**.	**Pseudo-F**	***p***	**Coeff**
Latitude	0.122	59.11	0.001	−9.42					0.179	99.17	0.001	12.42
Depth	0.165	68.44	0.001	24.97	0.020	16.06	0.001	8.40	0.014	11.58	0.001	−26.28
Temp	0.009	6.39	0.001	−72.16	0.006	5.29	0.001	−9.33	0.066	40.61	0.001	54.18
Sal	0.012	8.07	0.001	42.25			NS		0.014	12.55	0.001	−35.68
Oxygen	0.029	17.28	0.001	−18.58			NS		0.012	10.68	0.001	24.07
Alkalinity	0.008	5.10	0.001	14.31	0.004	3.13	0.030	8.18			NS	
DIC	0.005	3.62	0.001	−85.71	0.289	140.40	0.001	−16.07	0.197	84.94	0.001	53.93
Al (nM)			NS				NS		0.021	14.78	0.001	4.12
Cd (nM)	0.006	4.30	0.010	−26.26	0.005	4.08	0.010	21.01	0.006	5.52	0.004	27.53
Fe (nM)	0.005	3.35	0.025	−1.74	0.009	7.48	0.001	−2.58	0.004	3.40	0.016	0.46
Mn (nM)	0.045	25.55	0.001	10.28	0.062	44.39	0.001	9.90	0.003	2.87	0.024	−5.91
Ni (nM)	0.019	11.92	0.001	29.78			NS		0.020	15.67	0.001	−23.80
Pb (nM)	0.063	33.64	0.001	18.07	0.027	20.55	0.001	3.78	0.010	7.48	0.001	−13.39
Zn (nM)	0.005	3.30	0.001	−4.03			NS				NS	
Y (pM)	0.010	7.00	0.001	−16.42	0.007	5.87	0.020	−7.31	0.018	14.75	0.001	−0.66
La (nM)	0.025	15.89	0.001	7.51	0.093	52.10	0.001	6.96	0.005	4.27	0.009	5.46
Fluorescence	0.013	8.25	0.001	0.26	0.079	50.41	0.001	8.79	0.062	42.69	0.001	1.02
PO${}_{4}^{3-}$ (μM)	0.010	7.04	0.001	70.95			NS		0.005	4.79	0.005	-51.78
Si (μM)			NS		0.007	5.61	0.020	-4.89		9.29	NS	
NO${}_{2}^{-}$ (μM)			NS		0.006	4.66	0.016	4.16	0.013		0.001	-2.40
Cumulative	0.550				0.614				0.648			

*Pseudo-F and p-values were obtained by permutations (n = 999). Coeff: Coefficient for linear combinations of variables in the formation of dbRDA axis 1. As the basis of analyses, Bray-Curtis similarities were used for response variables. Oxygen, oxygen concentration (μM); DIC, dissolved inorganic carbon (μM)*.

### Negative vs. positive selection in LAC- vs. HAC-AOA clones

**Figure 7:**
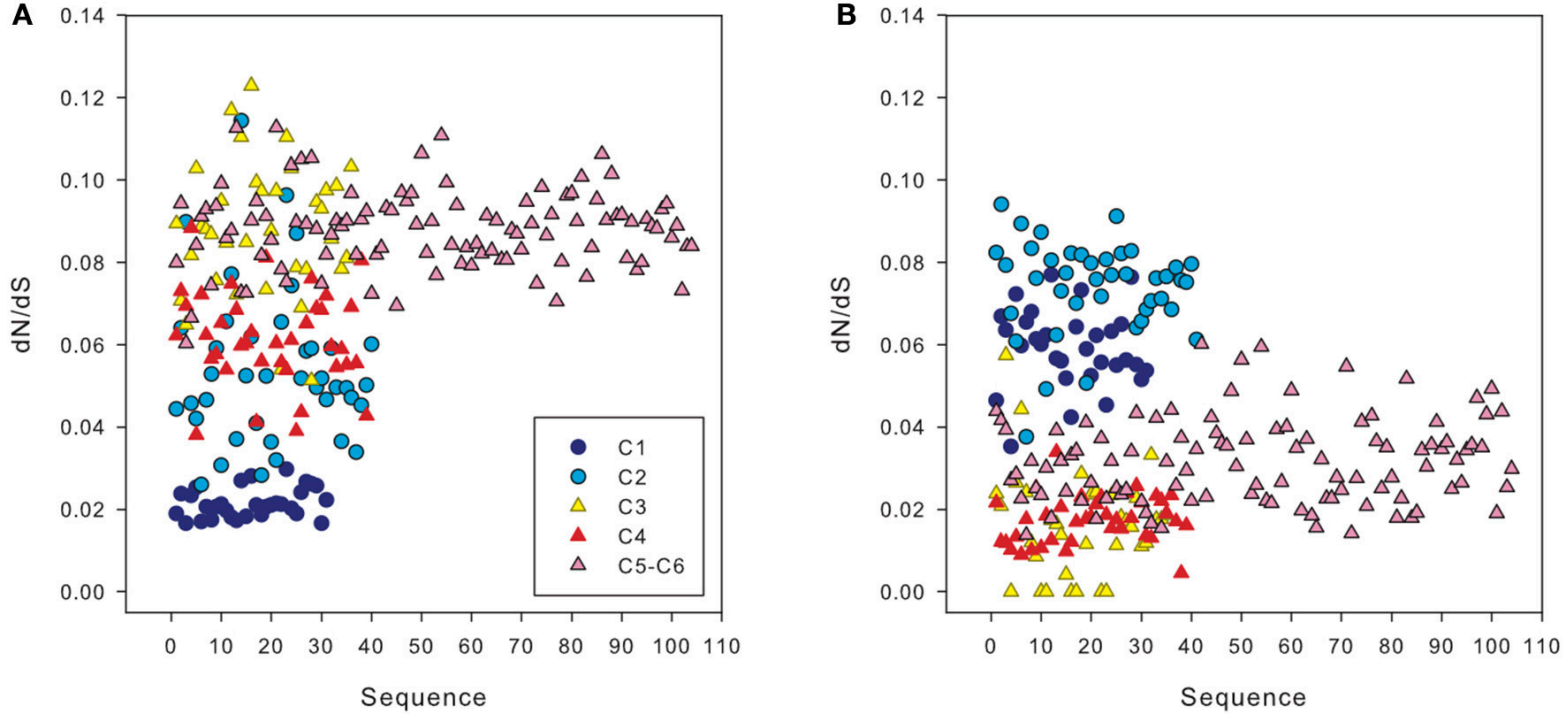
**dN/dS ratio between sequences from different clusters of archaeal *amo*A: of one representative sequence from cluster 2 vs. the sequences from other clusters (A) and of one sequence from cluster 3 vs. *amo*A sequences from other clusters (B), calculated using Ka/Ks calculator**. Cluster numbers (c1 to c6) correspond to the subclusters defined in Figure 3.

The ratio of non-synonymous to synonymous substitutions (dN/dS) obtained in our dataset was low, with an average of 0.07. However, when we compared *amo*A sequences from a clone belonging to clusters 1 and 2 (i.e., HAC-AOA) with sequences from clusters 3 to 6, we obtained a higher dN/dS ratio than comparing the same sequences to other clones from clusters 1 and 2 (Figure 7A). Similarly, sequences from clusters 3 to 6 had higher dN/dS ratios with sequences from clusters 1 and 2 than with other sequences from clusters 3 to 6 (Figure 7B).

## Discussion

### Factors driving the biogeography of AOA throughout the atlantic: the ecological significance of HAC- and LAC-AOA groups

The ubiquitous distribution of AOA is well documented in marine waters (Francis et al., 2005; Coolen et al., 2007; Herfort et al., 2007), with a few studies spanning over the entire water column to the deep ocean (Mincer et al., 2007; Yakimov et al., 2007; Agogué et al., 2008; De Corte et al., 2009; Beman et al., 2012). Since the description of two phylogenetically different water column clusters by Francis et al. (2005), several attempts to quantify different groups of AOA were conducted (Beman et al., 2008; Sintes et al., 2013; Smith et al., 2014; Villanueva et al., 2015). Our results support both the ubiquitous distribution of Thaumarchaeota in the ocean, as well as their differentiation into two clusters of AOA according to the prevailing environmental conditions in different oceanographic regions and depth layers.

Thaumarchaeota 16S rRNA gene and total *amo*A gene abundance in the gyral and tropical Atlantic agree with previous findings from the Pacific (Church et al., 2010) and Atlantic (Sintes et al., 2013). Moreover, the 16S rRNA gene abundance of Thaumarchaeota and HAC-AOA in Arctic and Subantarctic epi- to mesopelagic waters are in the range of abundances reported in Arctic and Antarctic coastal waters (Kalanetra et al., 2009). However, our study reports the distribution of Thaumarchaeota 16S rRNA gene, AOA and two AOA ecotypes from Arctic to Subantarctic waters and from epi- to bathypelagic depth at high resolution. HAC-AOA was dominant at high latitude provinces (ARCT, NADR, SANT) and in epipelagic waters at low latitudes in agreement with previous findings from the North Atlantic (Agogué et al., 2008; Sintes et al., 2013). In contrast, the LAC dominated at low latitude provinces (NAG, SATL, WTRA) and in bathypelagic waters, in agreement with recent findings in the deep northeast Pacific ocean (Smith et al., 2016). Thus, the distribution of these two clusters is related to the oceanographic regions described by Longhurst (2007).

The AOA community composition and distribution are influenced by diverse environmental factors (Erguder et al., 2009). Intuitively, ammonium concentration, the substrate for nitrification, is an important factor determining not only the AOA abundance but also ammonia oxidation rates (Christman et al., 2011). The distribution of ammonium concentrations reported in the literature (Woodward and Rees, 2001; Clark et al., 2008; Varela et al., 2008) seems to support the observed distribution of HAC and LAC, as previously discussed for the North Atlantic (Sintes et al., 2013). In the subpolar provinces, there is a larger phytoplankton bloom based on diatoms leading to higher export flux of organic matter, due to the larger cells, and the lower stratification (Koeve, 2002; Tremblay et al., 2002; Longhurst, 2007). Contrarily, in the gyre systems, small phytoplankton lead to lower export flux due to lower sinking rates, and at the same time stratification is higher. All this leads to lower export of organic N and consequently lower ammonium supply in the gyre regions. Yet, the concentration of ammonium at which the LAC ecotype outcompetes the HAC-AOA in oligotrophic waters is probably below or close to the detection limit of ammonium in seawater. *N. maritimus* expressed similar transcript levels for *amo*A under high (500 μM) and low (10 nM) ammonium concentrations (Nakagawa and Stahl, 2013), Moreover, both ecotypes actively transcribe the *amo*A gene down to the bathypelagic environment, where ammonia is under the detection limit of current methods (Smith et al., 2016). Together, these findings support the notion that the HAC cluster can still perform ammonia oxidation at ammonium concentration at or below the detection level of commonly applied spectrophotometric methods to determine ammonium concentrations. Further studies with improved methods to measure ammonium concentrations and/or ammonia oxidation in oligotrophic and deep waters are needed to determine the actual ranges of ammonia supply rates at which the different ecotypes can thrive.

However, other environmental factors might also play a role in the distribution of the different AOA ecotypes. Most of the environmental variables available from the Geotraces expeditions (Mawji et al., 2015) contributed to explain the variation of HAC- and LAC-*amo*A abundance, and also their relative abundance (ratio LAC/HAC). Latitude, depth, temperature, dissolved oxygen, nitrite, and salinity have been previously identified influencing the abundance and diversity of AOA (Francis et al., 2005; Herfort et al., 2007; Santoro et al., 2008; Abell et al., 2010; Biller et al., 2012; Pester et al., 2012; Sintes et al., 2015). These factors are also determining the distribution of the two ecotypes of AOA and the ratio LAC/HAC. Additionally, alkalinity, DIC, and all the trace elements tested explain to some degree the variation of these ecotypes in the Atlantic. Many trace elements are cofactors for enzymatic reactions and could potentially limit enzymatic reaction rates as has been suggested for copper and ammonia oxidizing Archaea (Jacquot et al., 2014). Currently, however, we cannot determine whether these trace elements exert some specific regulatory functions on metabolic processes in AOA.

### Insights into the ecotype differentiation based on oligotyping analysis

Oligotyping further supports the existence of different thaumarchaeal ammonia oxidizing ecotypes adapted to the environmental conditions prevailing in low vs. high latitude regions. Epipelagic and bathypelagic ecotypes, as well as polar vs. tropical ecotypes were distinguished according to the oligotype composition of the *amo*A gene clone libraries (Figure 5) and remarkably, also at the amino acid level (Figures 4, 6). Thus, the differentiation into these two ecotypes extends to the protein responsible for ammonia oxidation. Some amino acid substitutions are particularly significant, e.g., the change of glycine in position 65 and 77 (Figure 6) with alanine and valine, respectively. Glycine is often a highly conserved residue in protein families (Branden and Tooze, 1999) since it is essential for preserving the protein 3-D fold as it is normally present in turns (Lodish et al., 2003). Also, the substitutions between hydrophobic and polar residues, such as between alanine and serine, leucine and threonine, cysteine and alanine (Figure 6), can be sufficient to produce a change in the secondary structure of the protein (Cordes et al., 1999). Amino acids substitutions can result in diminished activity or loss of activity (Pakula and Sauer, 1989; Wang et al., 2004), as well as in increased affinity (Ricke et al., 2004). Indeed, even though the *amo*A subunit is assumed to be the active site of ammonia oxidation in Bacteria (Rotthauwe et al., 1997), it is not clear which subunit hosts the active site in Thaumarchaeota. For a closely related enzyme, the particulate methane monooxygenase (pmo), the *pmo*B subunit was identified as the active site (Balasubramanian et al., 2010; Lawton et al., 2014), and for another copper-containing membrane monooxygenase, the subunit C has been signaled (Liew et al., 2014). Thus, how the observed changes in amino acids may affect the functioning of the ammonia monooxygenase remains enigmatic.

Amino acid substitutions in the enzyme might lead to an adaptive increase in affinity toward the substrate due to the low ammonium concentrations in low latitudes and deep waters, to the transformation of the enzyme to catalyze the reaction on a (slightly) different substrate, or to the loss of the enzyme function due to a lower impact of ammonia oxidation on the Thaumarchaeota fitness when ammonium concentrations are too low to be efficiently used. In agreement with this latter possibility, recent findings on the distribution of AOA ecotypes, their transcription and nitrification rates in the Pacific ocean suggest that WCB (corresponding to our LAC) could access a wider range of substrates (Smith et al., 2016) as compared to WCA, and that only a fraction of the community oxidizes ammonia. Likewise, the changes in the enzyme could be neutral for the performance of the reaction (Pakula and Sauer, 1989). Consequently, further studies including ammonium concentration measurements and/or experiments with isolates representative of both groups should be conducted to better understand the mechanisms underlying the distribution and the changes in the protein sequences in both groups. Remarkably, only thaumarchaeotal representatives of the HAC-*amo*A cluster have been isolated thus far (Könneke et al., 2005; Santoro and Casciotti, 2011; Park et al., 2014; Qin et al., 2014) (Figure 3) indicating that more emphasis should be put on offering more realistic ammonium concentrations in culturing approaches than done hitherto.

### Selective pressure on archaeal ammonia oxidizers

It has been suggested that the existence of distinct archaeal *amo*A sequences in different environments could be explained by varying selective pressure on the function of the ammonia monooxygenase enzyme complex (Biller et al., 2012). The low ratio of non-synonymous to synonymous substitutions (dN/dS) obtained in our dataset, on average 0.07, implies a purifying (negative) selection on disadvantageous mutations of the gene in agreement with previous findings comparing AOA from different environments (Biller et al., 2012).

Weaker negative selection (thus stronger positive selection) on *amo*A sequences of the clusters 1 and 2 (HAC-*amo*A) vs. sequences of clusters 3–6 (LAC-*amo*A) (Figure 7) might be an indication of environmental changes selecting for functional differences in *amo*A (Biller et al., 2012). The environmental selection of different *amo*A gene clusters ultimately leads to niche specialization of these two AOA groups adapted to medium-high, on the one hand, and to low ammonia concentration environments (Sintes et al., 2013), on the other hand. However, the mechanisms responsible for this relationship between the two AOA clusters and ammonium concentrations are still unclear, and could comprise different affinity for ammonia, different ammonia permeases, or concentrating mechanisms (Sintes et al., 2013). Other possible causes for a weak negative selection might include a change in the impact of *amo*A to the overall fitness of Thaumarchaeota in these environments or the population size that could affect the efficacy of purifying selection (Biller et al., 2012). This latter hypothesis might explain the finding of weak correlations between nitrification rates and the deep cluster (Beman et al., 2008; Smith et al., 2014), and would agree with the mixotrophy (Qin et al., 2014) or heterotrophy of AOA (Teira et al., 2006; Li et al., 2015).

Taken together, our results indicate the existence of two main ecotypes of AOA in the ocean, which show a geographic distribution related to environmental conditions, extending the results of previous studies (Beman et al., 2008; Sintes et al., 2013; Smith et al., 2014; Villanueva et al., 2015) to the whole Atlantic Ocean. The in-depth analysis of the *amo*A gene and protein sequences conducted in this study not only supports these previous results but provides evidence for selection on the amo protein. The *amo*A amino acid sequence exhibits consistent differences between the two ecotypes, some of which can cause changes in the 3D-structure and activity of the enzyme. Although with the information available at the moment, we cannot determine the specific effect of these changes (e.g., increased affinity, loss of enzymatic function), the strong positive selection between the two ecotypes might indicate some degree of environmental selection leading to their niche specialization.

## Author contributions

ES and GH designed the work and wrote the study. ES, DD, and EH performed research. ES performed all the data analysis.

### Conflict of interest statement

The authors declare that the research was conducted in the absence of any commercial or financial relationships that could be construed as a potential conflict of interest.
