# Supplementary material for: Geographic Distribution of Archaeal Ammonia Oxidizing Ecotypes in the Atlantic Ocean
Source: Front Microbiol. 2016 Feb 9;7:77. doi: 10.3389/fmicb.2016.00077 (PMC4746290; doi:10.3389/fmicb.2016.00077)
Supplement: Supplementary file 1 [file Table1.DOCX]

**Supplementary Table 1.** Average ± SD, and minimum - maximum abundances (gene mL^-1^) of LAC-AOA, HAC-AOA, total AOA (AOA), and 16S rRNA gene from Thaumarchaeota (Thaum) and ratios LAC/HAC, and total AOA to 16S rRNA gene ratio (AOA/Thaum) for different depth layers (epipelagic, Epi, mesopelagic, Meso, upper bathypelagic, UpperBathy, lower bathypelagic, LowerBathy) in different oceanographic regions (ARCT, North Atlantic Arctic, NADR, North Atlantic Drift, NAG, North Atlantic Gyre, WTRA, Western Tropical Atlantic, SATL, South Atlantic Gyre, SANT, Subantarctic), and for the whole Atlantic transect.

|  |  | **LAC-AOA** | **HAC-AOA** | **AOA** | **LAC/HAC** | **Thaum** | **AOA/Thaum** |
| --- | --- | --- | --- | --- | --- | --- | --- |
| **ARCT** | **Epi** | 27±20 | 1146±571 | 1174±564 | 0.028±0.022 | 519±388 | 4.38±4.79 |
|  |  | 8-45 | 690-1980 | 700-1990 | 0.004-0.047 | 83-945 | 1.11-11.5 |
|  | **Meso** | 757±811 | 4151±4350 | 4903±4633 | 0.356±0.369 | 4061±3389 | 1.46±1.05 |
|  |  | 46-2710 | 323-13800 | 679-14800 | 0.014-1.102 | 303-9310 | 0.5-3.53 |
|  | **UpperBathy** | 1260±990 | 430±408 | 1691±1260 | 4.318±2.807 | 4552±3347 | 0.44±0.37 |
|  |  | 272-3070 | 73-1130 | 345-3730 | 1.336-8.504 | 691-9590 | 0.13-1.05 |
|  | **LowerBathy** | 839±980 | 389±486 | 1228±898 | 8.29±13.284 | 2817±1813 | 0.5±0.21 |
|  |  | 38-2620 | 77-1310 | 142-2690 | 0.246-33.938 | 173-5407 | 0.19-0.82 |
| **NADR** | **Epi** | 45±101 | 3693±4995 | 3746±5102 | 0.007±0.008 | 1640±2187 | 2.32±0.68 |
|  |  | 1-251 | 368-12600 | 369-12900 | 0.001-0.02 | 168-5300 | 1.63-3.29 |
|  | **Meso** | 501±423 | 3140±1918 | 3642±2139 | 0.352±0.625 | 5271±1698 | 0.69±0.33 |
|  |  | 24-1570 | 154-6340 | 504-7910 | 0.006-2.273 | 2480-8530 | 0.1-1.42 |
|  | **UpperBathy** | 805±510 | 448±463 | 1253±867 | 3.974±4.133 | 4321±2289 | 0.29±0.11 |
|  |  | 208-1770 | 51-1380 | 314-3150 | 0.623-13.642 | 1279-8670 | 0.17-0.46 |
|  | **LowerBathy** | 373±241 | 241±403 | 615±458 | 6.938±7.379 | 2071±981 | 0.36±0.31 |
|  |  | 119-1050 | 9-1500 | 128-1860 | 0.237-21.875 | 638-3560 | 0.05-1.22 |
| **NAG** | **Epi** | 22±63 | 738±1827 | 760±1826 | 0.974±1.809 | 578±876 | 0.77±0.63 |
|  |  | 0-262 | 0-7410 | 0-7430 | 0.001-6.209 | 1-3545 | 0.19-2.29 |
|  | **Meso** | 1384±1162 | 1824±3488 | 3210±3584 | 20.36±30.602 | 7636±5633 | 0.45±0.47 |
|  |  | 5-5230 | 5-15900 | 283-16700 | 0.005-107.692 | 578-27037 | 0.04-3.05 |
|  | **UpperBathy** | 583±291 | 52±95 | 635±342 | 84.95±124.558 | 1655±1028 | 0.54±0.45 |
|  |  | 87-1380 | 1-380 | 92-1760 | 1.599-531.348 | 271-4547 | 0.1-1.77 |
|  | **LowerBathy** | 404±224 | 10±13 | 414±234 | 62.496±42.558 | 1327±1026 | 0.46±0.32 |
|  |  | 31-971 | 1-66 | 33-1040 | 10.699-202.144 | 56-4188 | 0.09-1.44 |
| **WTRA** | **Epi** | 30±22 | 3275±5053 | 3312±5081 | 6.349±9.53 | 2812±3938 | 1.64±2.16 |
|  |  | 2-70 | 1-15000 | 10-15100 | 0.002-24.6 | 24-9687 | 0.17-5.82 |
|  | **Meso** | 7954±6592 | 717±1004 | 8671±7380 | 38.251±56.027 | 24685±33591 | 1.9±3.32 |
|  |  | 896-21800 | 5-3990 | 910-25800 | 4.956-261.905 | 345-109280 | 0.15-11.96 |
|  | **UpperBathy** | 1760±1389 | 35±35 | 1795±1423 | 121.631±162.897 | 11251±22477 | 0.6±0.54 |
|  |  | 346-4300 | 2-100 | 356-4390 | 36.042-476.19 | 198-77760 | 0.06-1.8 |
|  | **LowerBathy** | 971±768 | 20±13 | 991±779 | 57.164±60.519 | 5039±6099 | 1.21±2.75 |
|  |  | 2-2310 | 2-43 | 5-2340 | 0.995-282.258 | 2-21387 | 0.07-11.86 |

Supplementary Table 1. Cont.

|  |  | **LAC-AOA** | **HAC-AOA** | **AOA** | **LAC/HAC** | **Thaum** | **AOA/Thaum** |
| --- | --- | --- | --- | --- | --- | --- | --- |
| **SATL** | **Epi** | 5±3 | 25±23 | 30±24 | 2.807±5.783 | 245±451 | 0.4±0.49 |
|  |  | 2-11 | 0-66 | 6-68 | 0.036-17.305 | 15-1427 | 0.03-1.59 |
|  | **Meso** | 2804±1158 | 6117±6181 | 8918±5308 | 4.2±6.774 | 25822±17193 | 0.55±0.57 |
|  |  | 722-5030 | 164-19000 | 3350-19900 | 0.049-19.451 | 8565-60480 | 0.14-1.76 |
|  | **UpperBathy** | 1628±808 | 203±398 | 1831±1120 | 31.682±17.428 | 6745±2923 | 0.3±0.2 |
|  |  | 516-3780 | 12-1600 | 528-4480 | 1.763-51.542 | 2635-14837 | 0.13-0.75 |
|  | **LowerBathy** | 839±675 | 35±61 | 873±725 | 53.657±27.83 | 3146±1762 | 0.34±0.27 |
|  |  | 133-3690 | 2-301 | 135-3990 | 7.457-119.78 | 783-6272 | 0.09-1.22 |
| **SANT** | **Epi** | 266±639 | 22232±31134 | 22502±31707 | 0.015±0.017 | 7504±15742 | 7.63±4.73 |
|  |  | 4-1570 | 103-78000 | 107-79600 | 0-0.04 | 11-39573 | 2.01-15.32 |
|  | **Meso** | 3866±2439 | 26820±16762 | 30675±17259 | 0.213±0.215 | 13680±8283 | 10.87±26.13 |
|  |  | 546-9740 | 7660-56000 | 12300-58500 | 0.026-0.813 | 219-27413 | 1.05-85.08 |
|  | **UpperBathy** | 4493±1786 | 1493±1181 | 5990±2793 | 3.935±1.939 | 7443±3679 | 1.08±1.29 |
|  |  | 1940-7460 | 535-4020 | 2500-11500 | 1.856-7.647 | 1408-13168 | 0.42-4.25 |
|  | **LowerBathy** | 2099±1101 | 193±137 | 2290±1157 | 14.685±8.535 | 4593±2564 | 0.58±0.33 |
|  |  | 197-4490 | 16-539 | 213-4730 | 2.412-34.369 | 306-9451 | 0.35-1.85 |
|  |  |  |  |  |  |  |  |
| **Atlantic** | **Epi** | 53±225 | 3982±12421 | 4037±12618 | 1.857±4.934 | 1833±5842 | 2.13±3.18 |
|  |  | 0-1570 | 0-78000 | 0-79600 | 0-24.6 | 1-39573 | 0.03-15.32 |
|  | **Meso** | 2838±3899 | 5247±9865 | 8084±10745 | 14.898±32.64 | 13182±18058 | 1.79±8.12 |
|  |  | 5-21800 | 5-56000 | 283-58500 | 0.005-261.905 | 219-109280 | 0.04-85.08 |
|  | **UpperBathy** | 1501±1483 | 329±643 | 1830±2005 | 51.713±100.052 | 5338±8990 | 0.51±0.57 |
|  |  | 87-7460 | 1-4020 | 92-11500 | 0.623-531.348 | 198-77760 | 0.06-4.25 |
|  | **LowerBathy** | 881±879 | 91±206 | 972±936 | 43.174±41.89 | 2981±3097 | 0.56±1.12 |
|  |  | 2-4490 | 1-1500 | 5-4730 | 0.237-282.258 | 2-21387 | 0.05-11.86 |
|  |  |  |  |  |  |  |  |
| **Atlantic** | **all depths** | 1518±2542 | 2299±7547 | 3817±8248 | 30.485±58.606 | 6539±12180 | 1.15±4.78 |
|  |  | 0-21800 | 0-78000 | 0-79600 | 0-531.348 | 1-109280 | 0.03-85.08 |
